# A leaf-level spectral library to support high-throughput plant phenotyping: predictive accuracy and model transfer

**DOI:** 10.1093/jxb/erad129

**Published:** 2023-04-05

**Authors:** Nuwan K Wijewardane, Huichun Zhang, Jinliang Yang, James C Schnable, Daniel P Schachtman, Yufeng Ge

**Affiliations:** Department of Agricultural and Biological Engineering, Mississippi State University, Starkville, MS, USA; College of Mechanical and Electrical Engineering, Nanjing Forestry University, Nanjing, China; Jiangsu Co-Innovation Center of Efficient Processing and Utilization of Forest Resources, Nanjing Forestry University, Nanjing, China; Department of Agronomy and Horticulture, University of Nebraska-Lincoln, Lincoln, NE, USA; Center for Plant Science Innovation, University of Nebraska-Lincoln, Lincoln, NE, USA; Department of Agronomy and Horticulture, University of Nebraska-Lincoln, Lincoln, NE, USA; Center for Plant Science Innovation, University of Nebraska-Lincoln, Lincoln, NE, USA; Department of Agronomy and Horticulture, University of Nebraska-Lincoln, Lincoln, NE, USA; Center for Plant Science Innovation, University of Nebraska-Lincoln, Lincoln, NE, USA; Department of Biological Systems Engineering, University of Nebraska-Lincoln, Lincoln, NE, USA; Center for Plant Science Innovation, University of Nebraska-Lincoln, Lincoln, NE, USA; MPI of Molecular Plant Physiology, Germany

**Keywords:** Biochemical traits, camelina, extra-weighted spiking, high-throughput phenotyping, leaf hyperspectral reflectance, machine-learning, maize, partial least squares regression, sorghum, soybean, trait modeling

## Abstract

Leaf-level hyperspectral reflectance has become an effective tool for high-throughput phenotyping of plant leaf traits due to its rapid, low-cost, multi-sensing, and non-destructive nature. However, collecting samples for model calibration can still be expensive, and models show poor transferability among different datasets. This study had three specific objectives: first, to assemble a large library of leaf hyperspectral data (*n*=2460) from maize and sorghum; second, to evaluate two machine-learning approaches to estimate nine leaf properties (chlorophyll, thickness, water content, nitrogen, phosphorus, potassium, calcium, magnesium, and sulfur); and third, to investigate the usefulness of this spectral library for predicting external datasets (*n*=445) including soybean and camelina using extra-weighted spiking. Internal cross-validation showed satisfactory performance of the spectral library to estimate all nine traits (mean *R*^2^=0.688), with partial least-squares regression outperforming deep neural network models. Models calibrated solely using the spectral library showed degraded performance on external datasets (mean *R*^2^=0.159 for camelina, 0.337 for soybean). Models improved significantly when a small portion of external samples (*n*=20) was added to the library via extra-weighted spiking (mean *R*^2^=0.574 for camelina, 0.536 for soybean). The leaf-level spectral library greatly benefits plant physiological and biochemical phenotyping, whilst extra-weight spiking improves model transferability and extends its utility.

## Teaser

A spectral library (400–2500 nm) of maize and sorghum is developed to accurately estimate nine leaf properties, and is also effective in predictions for soybean and camelina using extra-weighted spiking.

## Introduction

The ability to collect phenomic data at sufficiently high resolution with low cost has been a key bottleneck in many basic and applied plant research fields (Furbank and Tester, 2011). With recent advances in imaging (particularly low-cost RGB imaging) and image processing, this bottleneck has been largely relieved for traits related to size, biomass, and growth (Fahlgren *et al.*, 2015; Ge *et al.*, 2016). However, the bottleneck remains a major challenge when studying a wide range of traits related to plant physiology, function, and biochemistry, such as leaf thickness, photosynthetic parameters, transpiration, and nutrient status. Conventional approaches to the quantification of these traits either entail a destructive process where leaf samples are collected and subjected to laboratory-based analysis procedures (such as mineral contents), or the use of specialized instruments for quantification (such as parameters related to photosynthesis). These analyses are time-consuming, expensive, and labor-intensive, and therefore are difficult or impossible to scale to the phenotyping of large, replicated studies of diversity panels, structured populations, breeding material, or multi-environment trials.

Leaf-level hyperspectral reflectance data in the visible, near-infrared, and shortwave infrared region (VIS-NIR-SWIR, combined from 400–2500 nm) have been used as a rapid and non-destructive method for plant analysis (Blackburn, 2007; Prananto *et al.*, 2020). Applications of VIS-NIR-SWIR hyperspectral data for estimating many leaf physiological and biochemical traits in the context of high-throughput phenotyping have recently been reported for maize (Yendrek *et al.*, 2017; Ge *et al.*, 2019), wheat (Silva-Perez *et al.*, 2018), sorghum (Chai *et al.*, 2021), and other crop species (Ely *et al.*, 2019). Promising results have been reported for leaf nutrient contents such as nitrogen and phosphorus (Ge *et al.*, 2019), leaf mass per area (or equivalently leaf thickness; Coast *et al.*, 2019), leaf physiological parameters related to photosynthetic capacity and gas exchange (Meacham-Hensold *et al.*, 2019), and metabolites (Vergara-Diaz *et al.*, 2020). These studies demonstrate the great potential of VIS-NIR-SWIR hyperspectral data for high-throughput analysis of leaf traits related to plant physiology, function, and biochemistry, as well as the detection of genotypic differences of phenotypic traits (Yendrek *et al.*, 2017), and ultimately enable genetic association analyses to elucidate the controlling genetic factors (Grzybowski *et al.*, 2021).

The clear advantage of VIS-NIR-SWIR lies in its rapid and non-destructive nature. The acquisition of leaf-level hyperspectral data can be done *in vivo* and takes several seconds at most, which is critically important for large-scale, in-field phenotypic data collection. An additional advantage of VIS-NIR-SWIR is its multi-sensing capability for integrating complementary information. Provided that relevant calibration models are available, many leaf traits can be quantified simultaneously using data collected from a single VIS-NIR-SWIR leaf scan. This ability to quantify multiple traits simultaneously further reduces the cost of experiments, increases measurement throughput, and facilitates the study of pleiotropy and genetic trade-offs between different traits.

Estimating leaf traits from VIS-NIR-SWIR data usually employs a data-driven approach where multivariate statistical models are calibrated from a set of samples that have been chemically analysed in the laboratory or with sophisticated physiological instrumentation. These multivariate models can be as simple as linear regression models using vegetation indices, but can also be more complex models based on machine-learning algorithms (Heckmann *et al.*, 2017; Ge *et al.*, 2019). This data-driven approach poses two potential problems for the widespread use of VIS-NIR-SWIR. First, the development of calibration models is expensive and usually requires a large number of samples to be analysed in the laboratory. Second, because the models are calibrated on a limited set of samples (a single species, few genotypes, and one or two environments), the performance of these models on other independent datasets is not guaranteed and can decline substantially relative to their initial performance. This second problem has been illustrated and discussed previously (see Yendrek *et al.*, 2017; Silva-Perez *et al.*, 2018). One potential solution to address the first problem is to establish a leaf-level VIS-NIR-SWIR spectral library comprising many samples with both laboratory data and spectral data that is widely available to the research community. Models can be calibrated from the samples in the library and used for other projects, which makes the use of VIS-NIR-SWIR more practical and economical. However, the use of spectral libraries makes the question of how models will perform on out-of-sample datasets even more pressing. One potential solution to the second problem of performance on external datasets is an approach called ‘spiking’.

Spiking has previously been used in soil spectroscopy to improve the accuracy of regional or national spectral models at local scales (Guerrero *et al.*, 2014; Barthès *et al.*, 2020). The core concept of spiking is to add a small number of ‘local’ samples from an investigator’s own experiment to the library samples to form a calibration sample set so that the model will perform better than those calibrated from the spectral library samples alone. This procedure often increases the accuracy of the predictions for the samples from the local experiment. The larger the number of local samples, the higher the accuracy gained by spiking. However, a large spiking set still increases the cost of the application due to the need for laboratory analysis. As an alternative, ‘extra-weighted spiking’ increases the statistical weight of the spiking set by adding several copies of it to match the number of samples in the original spectral library. This forces the calibration to fit for the extra-weighted samples (i.e. the local samples) better than without extra-weighing, thus leading to more accurate predictions (Guerrero *et al.*, 2014).

The objectives of this current study were three-fold. First, to assemble and report a leaf-level VIS-NIR-SWIR spectral library that was constructed from maize and sorghum leaves collected across multiple years and experimental conditions. Second, to evaluate two machine-learning approaches, namely partial least-squares regression (PLSR) and deep neural networks (DNN), to estimate nine leaf properties. And third, to investigate the utility of this spectral library for predicting the nine leaf properties of external datasets for maize, sorghum, soybean, and camelina with ‘extra-weighted spiking’. We hypothesized that spiking with samples from specific experiments could extend the use of this spectral library for different crop species.

## Materials and methods

### Construction of the VIS-NIR-SWIR spectral library

Maize (*Zea mays*) and sorghum (*Sorghum bicolor*) leaf samples were collected from a series of seven field and greenhouse experiments conducted between 2018–2020 at University of Nebraska-Lincoln (Table 1). Leaves were collected and measured at developmental stages spanning the late-vegetative to flowering. In flowering plants, the 2nd, 3rd, and 4th leaves from the top of the plant (i.e. the first three leaves below the flag leaf) were sampled, while in late-vegetative stage plants the three leaves below the most recently emerged leaf were sampled. A total of 2460 samples were included in the spectral library.

**Table 1.** Summary of samples used in the VIS-NIR-SWIR spectral library and the independent, external samples used to assess the performance of the spectral library

	Dataset	Species	Year	Environment	*n*	Notes
Library samples (*n*=2460)	1	Maize	2018	Greenhouse	260	Maize diversity panel (Flint-Garcia *et al.*, 2005)
	2	Maize	2018	Field	567	Maize diversity panel under normal low-nitrogen treatments (Flint-Garcia *et al.*, 2005)
	3	Maize	2019	Field	497	Maize diversity panel under normal and low-nitrogen treatments (Flint-Garcia *et al.*, 2005)
	4	Maize	2020	Field	247	Maize association panel (Mazaheri *et al.*, 2019)
	5	Sorghum	2018	Greenhouse	322	Sorghum association panel (Boatwright *et al.*, 2022)
	6	Sorghum	2019	Greenhouse	299	15 genotypes of grain, sweet, and energy sorghum
	7	Sorghum	2020	Field	268	Sorghum association panel under normal and low-nitrogen treatments (Boatwright *et al.*, 2022)
Independent, external test sets (*n*=445)	8	Soybean	2019	Greenhouse	126	Single variety (Thorne)
	9	Camelina	2020	Greenhouse	96	12 genotypes under normal and reduced irrigation treatments
	10	Maize	2018	Field	163	Unknown varieties
	11	Sorghum	2019	Greenhouse	60	Single variety (Tx430) under two-factor nitrogen and water treatments (two levels each, four combinations)

A benchtop spectroradiometer (LabSpec4, Malvern Panalytical Ltd.) with a contact probe accessory was used to measure the VIS-NIR-SWIR reflectance spectra of the leaf samples. The spectral range of this instrument is 350–2500 nm, and the spectral resolution is 3 nm from 350–1000 nm and 10 nm from 1000–2500 nm. The instrument resampled the raw measurement to an interval of 1 nm, giving 2151 data points at every nm for each raw spectrum. The effective measurement area of the contact probe was 10 mm in diameter. Three spectral readings were taken at the tip, middle, and basal sections of the leaf (avoiding the mid-rib for maize and sorghum), and the nine readings were averaged as a final measurement to minimize the effects of within-leaf heterogeneity. Spectral readings were taken from the adaxial side of the leaves. A white panel with 99% reflectance (Spectralon^®^, Labsphere Inc.) was used every 15 min to keep the instrument well-calibrated during data acquisition. A dark panel (mean 2% reflectance) was kept under the scanning area of the leaf to prevent backscattering of the transmitted light through the leaf.

Laboratory data were collected for the following leaf traits: chlorophyll content (CHL), leaf mass per area (LMA), leaf water content (LWC), and the nutrient contents of nitrogen (N), phosphorus (P), potassium (K), calcium (Ca), magnesium (Mg), and sulfur (S). A handheld leaf chlorophyll meter (MC-100, Apogee Instruments) was used to measure CHL with its built-in calibrations for respective crop species. Leaf area (LA) was measured with a leaf area meter (LI-3100, LI-COR Biosciences). After the fresh weight (FW) of the leaf was recorded, it was placed in an oven at ~50 °C and dried over 72 h to a constant weight, at which point the dry weight (DW) was recorded. LWC (%) was derived as (FW–DW)/FW ×100. LMA (g m^–2^) was derived as DW/LA. Dried plant samples were ground, homogenized, and analysed for nutrient contents. N was determined using the Dumas method with a LECO FP428 nitrogen analyser (AOAC method 968.06). Other nutrients were determined using microwave nitric acid digestion followed by inductively coupled plasma spectrometry (AOAC method 985.01). All nutrient contents were expressed on a percentage of dry matter basis.

Independent test sets were collected from four species: maize, sorghum, soybean (*Glycine max*), and camelina (*Camelina sativa*; Table 1). The inclusion of soybean and camelina allowed us to evaluate the performance of the spectral library on different species. Both the laboratory leaf property data and VIS-NIR-SWIR spectral data for the four independent test sets were collected in the same way as the library data, with two distinctions. First, CHL and LMA were not measured for the camelina set because the leaf chlorophyll meter and the leaf area meter were not available. Second, because soybean and camelina have smaller leaves, the three spectral measurements on each leaf were made on a less spread-out area (in contrast to maize and sorghum on the tip, middle, and base section of the leaf).

### Model calibration, extra-weighted spiking, and validation

Before any model calibration for VIS-NIR-SWIR, wavelength averaging with a window size of 10 nm was implemented on all spectra to reduce the dimensionality and decrease the computational load. We investigated two machine-learning approaches to build the calibration models: partial least-squares regression (PLSR) and deep neural networks (DNNs). We selected these two techniques to represent a linear, less computationally demanding, more interpretable, and most frequently used method (PLSR) and a non-linear method that is reported to produce superior accuracies with spectral libraries (DNN; Viscarra Rossel and Behrens, 2010; Wijewardane *et al.*, 2016, 2018; Ng *et al.*, 2019).

PLSR is a conventional and commonly used spectroscopic modeling technique that implements an algorithm similar to principal component analysis to reduce predictors (i.e. the number of wavelengths) to a few latent variables. Unlike principal component analysis, construction of latent variables in PLSR considers the response variable (i.e. the property of interest) to ensure highest correlations. A linear model is then fitted between the response variable and latent variables (Helland, 2004).

DNNs consist of layers of nodes operating as non-linear summing devices similar to biological neurons. Nodes in each layer are connected to the nodes in the adjacent layers through weights that are optimized iteratively to produce the best-fitting model. These weights are adjusted by back-propagation where learning error is propagated back to the previous layers. Unlike PLSR, DNNs are not interpretable, but are considered effective when signal-to-noise ratio is low (Rumelhart *et al.*, 1988; Dayhoff and DeLeo, 2001; Hastie *et al.*, 2009).

Data analysis and plotting were implemented in Python 3.8 with the following libraries: scikit-learn (Pedregosa *et al.*, 2011), pandas (McKinney, 2010), NumPy (Stéfan van der Walt *et al.*, 2011), and Matplotlib (Hunter, 2007).

Our goal was to find the approach that gave the best overall performance to estimate the leaf traits. The entire library was used for model calibration with 10-fold cross-validation to avoid model overfitting and to identify the best tuning parameters. For PLSR, models having as many as 30 latent variables were considered. For DNNs, hidden layer sizes (5, 10, 15, and 20) and L2 penalty (0.005, 0.01, and 0.03) with the activation function ‘relu’ were used as the tuning parameters. The best tuning parameters were obtained considering the lowest cross-validated root mean-squared error (RMSE_CV_) in 10-fold cross-validation. The resulting best tuning parameters were then used to build a final model for each modeling technique using the whole dataset.

The steps to test the ‘extra-weighted spiking’ method to improve the model performance using the spectral library were as follows. Twenty samples from each external dataset were randomly selected as the ‘spiking’ sets. The remaining samples of each external dataset were considered as test sets. Three methods of model calibration and testing were compared. First, the models calibrated from the library were directly applied to the test sets. Secondly, models calibrated from the spiking sets (i.e. the 20 samples randomly drawn as the calibration set) were used to predict for the test sets. Third, we investigated how ‘extra-weighted spiking’ could improve the predictive performance for the external, independent sets by adding spiking sets to the library to form an augmented set. To increase the weight of the spike samples relative to the library (20 versus 2460), the spike samples were replicated 123 times such that the numbers of the two groups were the same. The method is therefore also referred to as ‘spiking with extra weight’. The augmented set (or the spiked library) was then used to develop the calibration models and validated on the test sets.

The impact of the size of the spiking set on the model transferability was further investigated by recalibrating and validating the models with spiking sets of different sizes. For this analysis, the soybean dataset was used as the validation set since it was a species not included in the spectral library and all the leaf properties had been measured. First, a randomly selected 50 samples from the soybean dataset was set aside to be used for spiking. From this spiking dataset, different number of samples (10, 20, 30, 40, 50) were randomly selected and used for PLSR model calibration with extra-weighted spiking. Each model was then used to predict for the remaining soybean samples. All the models were evaluated by calculating *R*^2^ (coefficient of determination), the root mean-squared error (RMSE), bias, and ratio of performance to deviation (RPD).

## Results

### Comparison of the library samples and the external independent test samples

The plant spectral library used in this study consisted of seven datasets collected from different field and greenhouse experiments from 2018–2020. There were four maize datasets (*n*=1571) and three sorghum datasets (*n*=889) making a total of 2460 leaf spectra with associated ground-truth information. The external test sets included four different datasets of soybean, camelina, maize, and sorghum, totaling to 445 plant spectra (Table 1). Note that the spectral library did not include any soybean or camelina spectra, providing us with the opportunity to evaluate how successfully the library could be employed for other non-grass species.

**Fig. 1.**
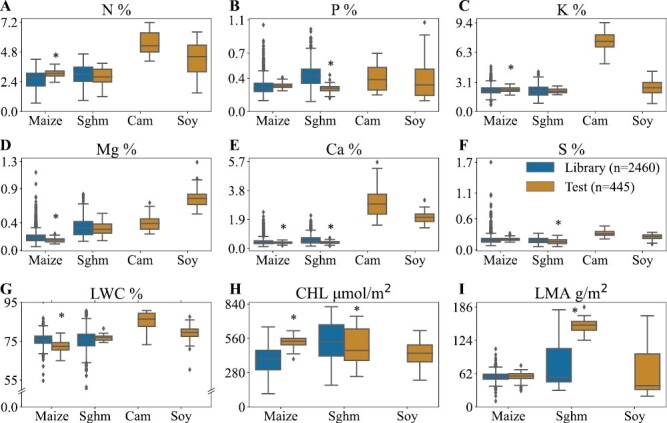
Variations in measured leaf properties among maize, sorghum (Sorg), camelina (Cam), and soybean (Soy) and between the spectral library and external test datasets for percentage contents of (A) N, (B) P, (C) K, (D) Mg, (E) Ca, and (F) S, (G) leaf water content (LWC), (H) chlorophyll content (CHL), and leaf mass per area (LMA). No data were obtained for CHL and LMA for camelina because the leaf chlorophyll meter and the leaf area meter were not available. The bottom, middle, and top lines of the boxes indicate the first quartile (Q1), median (Q2), and third quartile (Q3) of the data. Whiskers indicate the range of the data excluding outliers, which are identified as data points laying outside of Q1–1.5(Q3–Q1) and Q3 + 1.5(Q3–Q1). Significant differences between the library and the test set samples within a species were determined using ANOVA followed by Tukey’s Honestly Significant Difference: *P<0.05.

The distribution of the nine leaf properties for the spectral library and the independent external test sets are shown in Fig. 1 (numerical values are provided in Supplementary Table S1). The library and external test sets showed significant differences in maize for N, K, Mg, Ca, LWC, and CHL, and in sorghum for P, Ca, S, and LMA. When considering all four species, the library and the external test sets were significantly different from each other across all the properties except for the P content. .

**Fig. 2.**
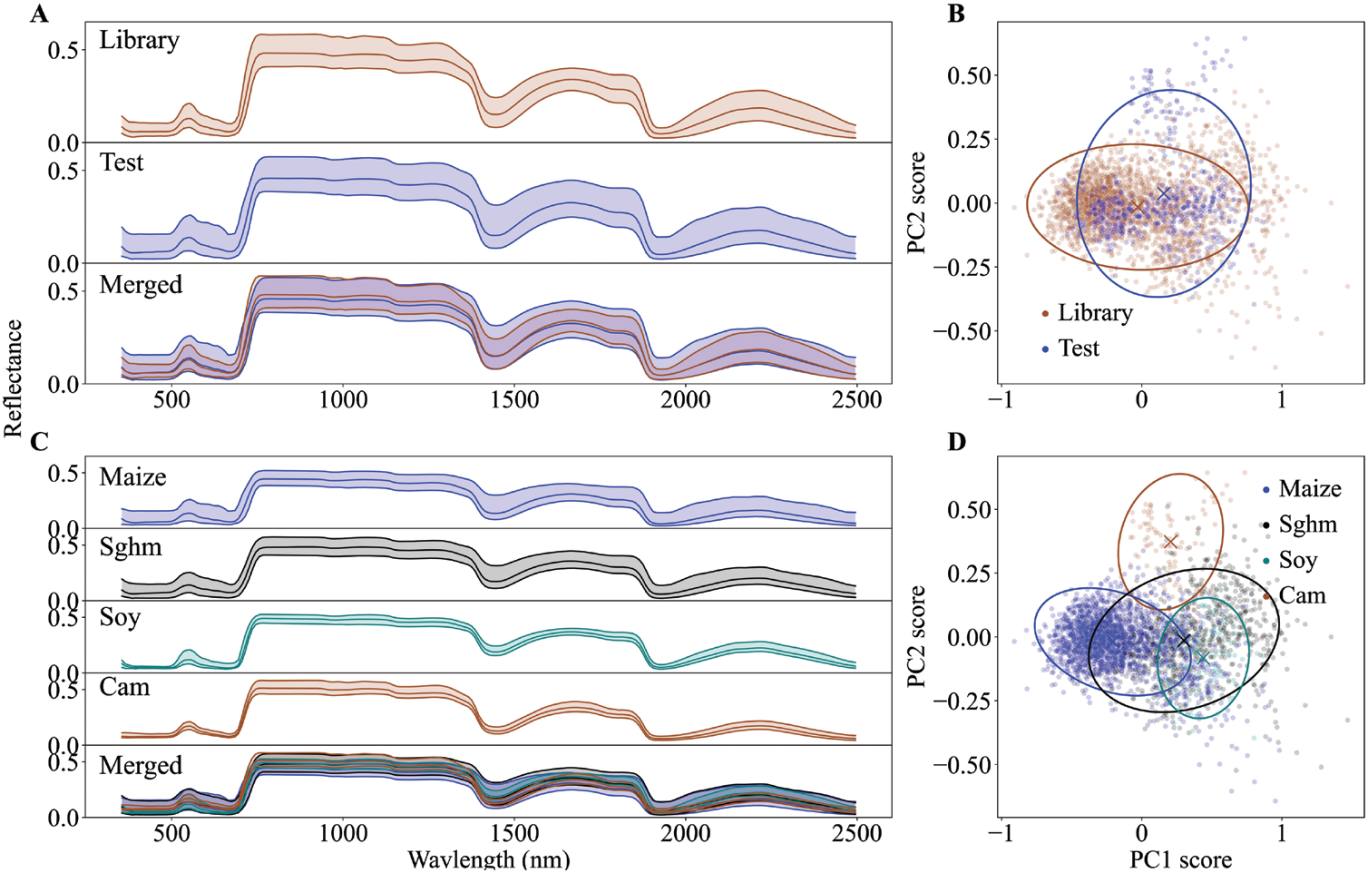
Spectra of the library, external test datasets, and the different species in the wavelength domain and the principal components space for maize, sorghum (Sorg), camelina (Cam), and soybean (Soy). (A) Spectra of the library and test datasets, with the two superimposed below (Merged), and (B) principal components analysis of the datasets. (C) Spectra of the datasets for the individual species, and all species superimposed below, and (D) principal components analysis of the species. The spectra show the mean and 95% confidence intervals, and the 95% ellipses are shown for the principal components.

The spectra of the different datasets and species, and their 95% confidence ellipses in principal component (PC) space are shown in Fig. 2, and statistical comparisons of variances and means in PC space among the different datasets and species using Levene’s and Hotelling’s *T*^2^ tests are given in Table 2 (Hotelling, 1992; Levene, 1960). There was a prominent spectral difference between the spectral library and the external test sets in both wavelength domain and PC space (Fig. 2A, B, Table 2). Compared to the spectral library, the external test datasets had a broader spectral variation in the wavelength domain, which could have resulted from the fact that the spectral library consisted of only two similar species while the external datasets had four different species with more diversity in terms of leaf morphology, physiology, and biochemistry. Both maize and sorghum are monocots while soybean and camelina are eudicots, hence differentiating the library and external test sets. Camelina stood out as a different spectral group in the PC2 space when compared to the other species. Maize showed less spectral overlap with camelina and soybean while more with sorghum (Fig. 2D).

**Table 2.** Statistical comparisons of variances and means in principal component space among the different datasets

Comparison	*P*-value			Conclusions
	Levene’s test		Hotelling’s *T*^2^ test	
	PC1	PC2		
Library/Test	<0.001	<0.001	<0.001	Different variances, different centers
Maize/Sorghum	<0.001	<0.001	<0.001	Different variances, different centers
Maize/Soybean	<0.001	0.003	<0.001	Different variances, different centers
Maize/Camelina	0.005	<0.001	<0.001	Different variances, different centers
Sorghum/Soybean	<0.001	0.273	<0.001	Different variances for PC1 only, different centers
Sorghum/Camelina	<0.001	0.968	<0.001	Different variances for PC1 only, different centers
Soybean/Camelina	0.351	0.333	<0.001	Same variances, different centers

### Comparison of modeling techniques

We compared PLSR and DNN using the cross-validation *R*^2^ value and the RMSE to identify the best modeling technique for estimating the different leaf properties using the spectral library. As shown in Table 3, the cross-validation *R*^2^ (*R*^2^_CV_) across all nine leaf properties varied from 0.36–0.92. PLSR showed higher accuracies than DNN except for LMA. Cross-validation RMSE (RMSE_CV_) showed a similar pattern, confirming the superiority of the PLSR models. Similar observations of superior performance from PLSR models compared to other machine-learning approaches have been reported previously for hyperspectral remote-sensing data (Siegmann and Jarmer, 2015; Krishna *et al.*, 2019). PLSR is a linear modeling technique while DNN is non-linear. We speculate that the inherent relationships between the leaf properties and spectra considered here are linear and that the non-linear modeling technique could capture the subtle noise components, thus causing degraded model performances. Because PLSR showed the best modeling performance, further analysis with ‘extra-weighted spiking’ and testing on the independent test sets were based on this technique.

**Table 3.** Cross-validation statistics of the two modeling techniques for the nine leaf traits using the VIS-NIR-SWIR spectral library

Leaf trait	Statistic	Modeling technique	
		PLSR	DNN
N (%)	RMSE_CV_	0.282	0.297
	*R* ^2^_CV_	0.842	0.825
P (%)	RMSE_CV_	0.085	0.093
	*R* ^2^_CV_	0.577	0.489
K (%)	RMSE_CV_	0.337	0.364
	*R* ^2^_CV_	0.517	0.437
Mg (%)	RMSE_CV_	0.062	0.089
	*R* ^2^_CV_	0.759	0.498
Ca (%)	RMSE_CV_	0.136	0.157
	*R* ^2^_CV_	0.742	0.667
S (%)	RMSE_CV_	0.06	0.072
	*R* ^2^_CV_	0.558	0.355
LWC (%)	RMSE_CV_	3.085	3.152
	*R* ^2^_CV_	0.437	0.41
CHL (μmol m^–2^)	RMSE_CV_	40.178	40.625
	*R* ^2^_CV_	0.92	0.918
LMA (g m^–2^)	RMSE_CV_	9.43	7.558
	*R* ^2^_CV_	0.841	0.898

PLSR, partial least-squares regression; DNN, deep neural networks. RMSE_CV_, root mean-squared error of cross-validation; *R*^2^_CV_, coefficient of determination of cross-validation. LWC, leaf water content; CHL, leaf chlorophyll content; LMA, leaf mass per area,

Among the nine leaf properties studied, CHL, LMA, and N were estimated most successfully, with *R*^2^_CV_>0.84. Mg and Ca were also estimated satisfactorily, with *R*^2^_CV_ of ~0.75. P, K, and S were estimated moderately well, with *R*^2^_CV_>0.5. LWC was the only property modeled with *R*^2^_CV_<0.5. The better model performance of CHL, LMA, and N from leaf hyperspectral data is in agreement with several other studies (Yendrek *et al.*, 2017; Silva-Perez *et al.*, 2018; Coast *et al.*, 2019). Chlorophylls have strong absorption peaks in the blue and red regions of the spectrum, resulting in a successful PLSR model to quantify its concentration. Leaf N can be successfully modeled primarily for two reasons. Firstly, about half of the N in fresh leaves is found in chlorophyll molecules; thus, a successful PLSR spectral model of CHL would also indicate a good model of N due to this association. Secondly, the other N in fresh leaves is primarily found in proteins and amino acids, which contain amide bonds that have absorption bands in the SWIR region ( 1550–1750 nm). LMA is a measure of leaf thickness, which effectively determines the path length of the hyperspectral light energy (thicker leaves have longer path lengths) and therefore results in a satisfactory PLSR model. Similarly, N, P, and S produce covalent bonds with carbon compounds (nucleic acids, sugar-phosphate intermediates, phospholipids, co-enzymes, sulfolipids, and amino acids) that are often present in photosynthetic complexes and absorb light in the VIS-NIR-SWIR region, which leads to spectral signatures that can be used for estimations (Vance *et al*., 2003; Benning *et al.*, 2008). The other leaf macronutrients that we examined, K, Ca, and Mg, often exist as free ions in living plant tissues and do not produce active spectral absorption features in the VIS-NIR-SWIR region. However, they do bond electrostatically or as ligands to larger carbon-containing compounds, and therefore can be indirectly derived as secondary compounds using spectroscopy (Hepler and Winship, 2010; Nieves-Cordones *et al.*, 2016; Pandey *et al.*, 2017). For example, Mg is a constituent of chlorophyll molecules and actively participates in the photosynthetic process (Palta, 1990), which can be correlated with the chlorophyll content of the leaf.

### Improvements by extra-weighted spiking

**Fig. 3.**
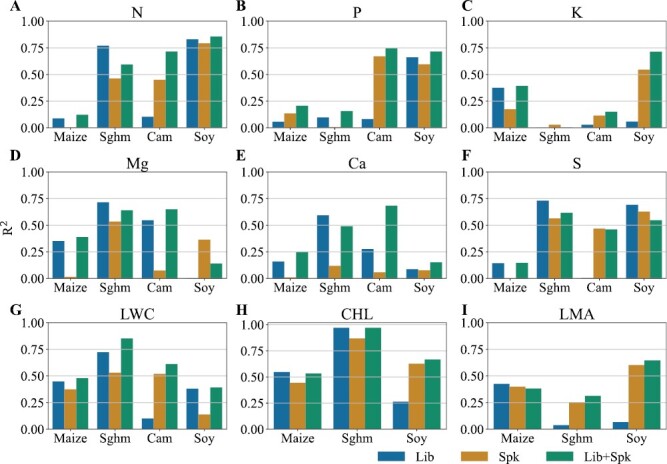
Values of *R*^2^ for maize, sorghum (Sorg), camelina (Cam), and soybean (Soy) for different leaf traits using the partial least-squares regression model calibrated on the spectral library only (Lib), the spike set only (Spk), and the extra-weighted spiked library (Lib+Spk). (A–F) percentage contents of (A) N, (B) P, (C) K, (D) Mg, (E) Ca, and (F) S. (G) Leaf water content (LWC), (H) chlorophyll content (CHL), and leaf mass per area (LMA). No data were obtained for CHL and LMA for camelina because the leaf chlorophyll meter and the leaf area meter were not available.

**Fig. 4.**
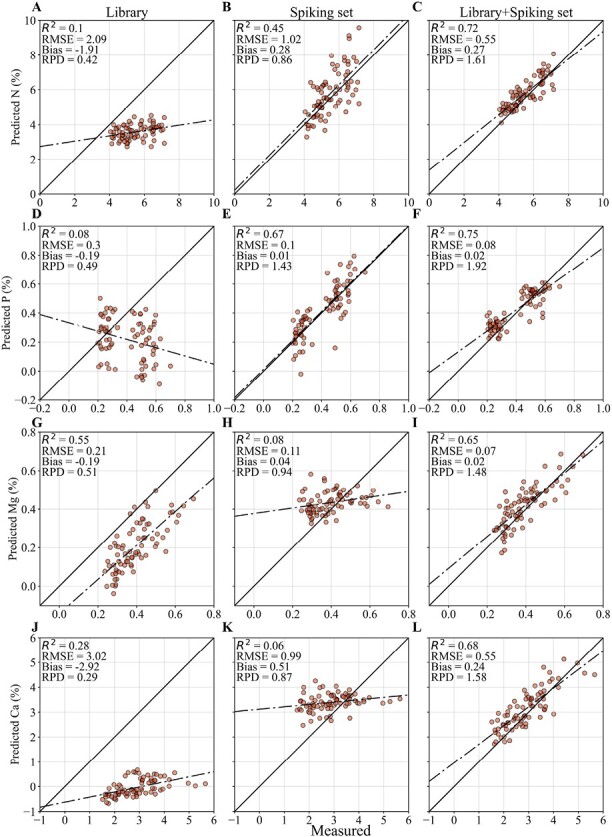
Prediction scatterplots for selected nutrient contents of camelina test samples (*n*=76) using the partial least-squares regression model calibrated on the spectral library only (Lib), the spike set only (Spk), and the extra-weighted spiked library (Lib+Spk). (A–C) N, (D–F) P, (G–I) Mg, and (J–L) Ca. RMSE, root mean-squared error; RPD, ratio of performance to deviation.

Independent validation on an external dataset is one of the most robust ways to assess likely model performance in real-world applications (Siegmann and Jarmer, 2015). In this study, we used datasets from the four different species maize, sorghum, camelina, and soybean as the external datasets to validate the model calibrated on the spectral library. Again, the spectral library consisted of data only from maize and sorghum. The spectral library was spiked (with extra-weight) using 20 samples from each external test set. Values for *R*^2^ and some selected prediction plots are shown in Figs 3 and 4 (complete summary statistics for all the model testing schemes and the leaf properties are provided in Supplementary Table S2).

Most of the models for the different leaf properties failed to show good predictions for the external datasets for any species using the spectral library models (Fig. 3). This might be due to the spectral differences between the library and test sets, as shown in Fig. 2B. Surprisingly, despite the fact that the spectral library incorporated large quantities of maize and sorghum data, the models were not able to accurately predict most of the properties of maize (N, P, K, Mg, Ca, S, LWC, and LMA) and some of the properties of sorghum (P, K, LMA). We speculate that this might be due to the differences in varieties and/or the treatments used in the specific experiments that we studied (Table 1). In addition, the variation of the spectral library may have been insufficient to calibrate accurate models even for maize and sorghum (see Fig. 1). The models built using only the spike sets also did not perform well, obviously due to the small number of samples (*n*=20) that was not enough to effectively capture the spectra–property relationships. However, when the spectral library was spiked with extra-weight, the models often showed better performances compared to the other two schemes. This was due to the introduction and equalizing of the local variability (i.e. variability in the external dataset) with the inherent variability of the spectral library through extra-weighing, which provided a balanced calibration dataset for the models to capture both global and local variations and hence increased the model robustness. For maize, a notable improvement was observed for P, while most of the other properties showed some increases in *R*^2^. The highest improvement due to spiking for sorghum was observed with LMA, while the other properties showed only marginal or no improvements. This was not surprising since the spectral library already consisted of maize and sorghum samples, which sufficiently captured the spectral variabilities for the models. Out of all the species tested, the most prominent improvements were observed for camelina, where all the properties measured showed enhanced *R*^2^, with six showing >100% improvement. Similarly, soybean showed >100% improvements for K, Mg, CHL, and LMA. Both camelina and soybean were not included in the original spectral library; regardless of this, extra-weighted spiking substantially enhanced the model performance for these datasets. This is noteworthy, because the spiking incorporated the spectral variability of a completely different species into the original calibration dataset, confirming the ability of spiking to successfully integrate new and unseen variability into the original dataset. In essence, spiking was effectively able to capture the local variabilities of the external datasets through the spike set to increase the model robustness for the species outside the spectral library. Similar improvements were observed for other statistics as well (Supplementary Table S2).

**Fig. 5.**
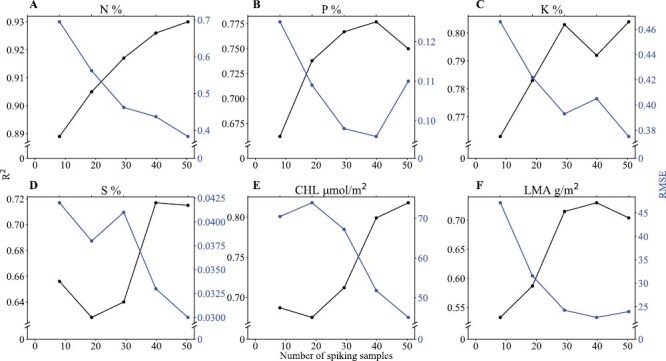
Effects of using different numbers of samples in the spiking set on the prediction performance (*R*^2^ and RMSE) of the partial least-squares regression model for leaf traits in soybean. (A–D) Percentage contents of (A) N, (B) P, (C) K, and (D) S. (E) Chlorophyll content (CHL and (F) leaf mass per area. RMSE, root-mean squared error.

The changes in model *R*^2^ and RMSE with increasing number of samples in the spiking set for the leaf properties is shown in Fig. 5 (with a threshold of *R*^2^>0.7). It can be seen from this analysis that there was a general increase in *R*^2^ and decrease in RMSE as the size of spiking set increased from 10 to 40, and the improvement started to level off toward a size of 50. This result was expected as a higher number of spiking samples would represent the external, independent dataset better, therefore leading to better model performance with the spiked library. However, when the spiking set reaches a certain size (40 in our analysis), continuing to include more samples will no longer lead to substantial increases in model performance. When deciding what an appropriate size for the spiking set should be, we consider not only model performance but also the practical cost, as each sample used for spiking will need to be analysed in the laboratory.

## Discussion

### A large spectral library benefits phenotyping of leaf physiological and biochemical traits, but the initial cost of its construction can be high

Spectral libraries such as the one reported in this study are a critical requirement towards implementing VIS-NIR-SWIR as a rapid, low-cost operational tool for phenotyping leaf physiological and biochemical traits. There have been a significant number of studies reporting promising results from the application of VIS-NIR-SWIR to quantify plant traits. These studies have all followed a traditional paradigm where hyperspectral models are calibrated and tested on data from the specific studies, and application of models on external, independent datasets has been rare. As noted above, collecting ground-truth data for hyperspectral modeling is typically the most expensive part of a study. It is neither economical nor practical to collect such data for a large number of samples to calibrate a model, and it is also wasteful if these data are just used once for a particular project. Plant leaf spectral libraries can effectively address this challenge by providing the necessary samples for model calibration while avoiding the need for collecting a large number of samples for ground-truthing during the field experiments, and this will lead to significant savings in both cost and time.

Ideally, the spectral libraries should span wide ranges of both genetic diversity and experimental conditions to ensure their applicability under wider settings, and hence include different species, physiological conditions (water, nutrients, temperature, and biotic stresses), growth stages, geographical regions, and environments (greenhouse, field, temperate, tropical). Such an all-inclusive spectral library will ensure robust model deployment. The spectral library developed in this paper can be dynamically grown through the addition of new spectra from laboratory chemical data. Thus, over time this spectral library can potentially become very large and diverse (e.g. including tens of thousands of samples and numerous species and experimental conditions), and provide useful training sets and prediction models to estimate many leaf properties rapidly and accurately. The ultimate goal is to compile a spectral library that can be used to build robust models without excessive cost to serve diverse applications including phenotyping, sensing, modeling, and precision agriculture.

The initial development of such a leaf spectral library can require large investments due to the need for a high number of samples and their associated laboratory work. Indeed, the scope of the spectral library could be well beyond the capacity of individual research laboratories. Here, one effective solution is to implement a community-based approach where researchers conducting similar work can contribute their data to build a central spectral library, and in turn they can get access to the whole library with a diverse sample set. The soil spectroscopy community has already initiated such an effort in the ‘Soil Spectroscopy 4 Global Good’ project (https://soilspectroscopy.org/), where the community contributes to building an open spectral library. A similar effort for plants could potentially contribute to building an equivalent large spectral library for the benefit of the plant science community. However, such collaborative spectral libraries can pose multiple challenges, and need a pragmatic approach in terms of such things as data ownership, privacy, standardization, operational costs, maintenance, and the high computational requirements for model calibration.

### Extra-weighted spiking is an effective strategy for improving model transferability and robustness from the spectral library

Another key challenge with the use of large spectral libraries is model transferability. That is, models calibrated on one set of samples often perform sub-optimally when applied on a different dataset; for example, from different years, experimental conditions, or crop species. This poor model transferability represents a limitation for the practical value of VIS-NIR-SWIR spectral libraries. Extra-weighted spiking is one methodology that can be used to enhance the model accuracy of the spectral library, and has been previously demonstrated in soil spectroscopy (Guerrero *et al.*, 2014), but not in plant spectroscopy. Due to a lack of variation for a plant trait in experiments from which a given spectral library has been derived, the model calibrated on the library alone may not yield satisfactory predictions. Including some local variability (i.e. a low number of samples from outside the spectral library) can improve model accuracy in situations where creating a calibration dataset solely from the local samples would be prohibitive in terms of cost and time. As can be seen from this study, increasing the number of samples in the spiking set can effectively increase the model transferability (i.e. model robustness) by incorporating more local variability into the calibration dataset (Figs 3, 4). However, increasing the number of samples in the spiking set requires more local samples to be collected, processed, scanned, and analysed, which can drastically increase the labor and cost. Therefore, the number of spiking samples is a trade-off between the cost of additional sample analysis and the effectiveness of transferability (the robustness) of the library models.

From our results, it was evident that even with a low number of spiking samples (*n*=20), the transferability of the models was enhanced (Fig. 5). This can broaden the applicability of our spectral library and reduce the cost of operation by enabling accurate and robust model calibrations for different crops under different settings (field, greenhouse, different stresses) for diverse applications. In such scenarios, the model accuracy could be further enhanced by the use of stratified random sampling, application of the Kennard–Stone algorithm, or use of Latin Hypercube Sampling instead of random sampling in the spectral space to account for diverse conditions in the external dataset.

Regardless of the model improvements that result from extra-weighted spiking, this technique significantly increases the number of samples in the calibration datasets. Indeed, it exactly doubles the number of calibration samples, which will increase the computational demand of the model calibration, especially when machine-learning techniques are used. This will require high-performance computing resources to be available; otherwise, low computationally demanding modeling techniques such as PLSR could be implemented for model calibration.

### A leaf spectral library is a key component to operationalize field-based hyperspectral phenotyping

With the advancement of sensor and robotic technologies and their adoption within the plant phenotyping community, the cost of obtaining hyperspectral reflectance data from leaves will continue to decline. There are a number of off-the-shelf, portable spectrometers that can be used in the laboratory and field environments (Prananto *et al.*, 2021). New developments have emerged such as a dedicated hyperspectral plant leaf scanner (Wang *et al.*, 2020) and the integration of a hyperspectral sensor with a robotic manipulator to automate data acquisition (Atefi *et al.*, 2019). However, hyperspectral data alone are of limited use unless they can be used to estimate leaf traits related to important plant processes and conditions. In this sense, the spectral library and the models calibrated from the library samples serve as an essential link between the sensor/raw hyperspectral data and the traits of interest. The library can also be incorporated as a software component to the hyperspectral sensors, such that leaf traits can be estimated in real time after a hyperspectral scan is acquired.

In recent years, hyperspectral measurements have been used as a tool to replace conventional destructive sampling and rapidly estimate plant biochemical and physiological traits, thereby enabling breeders and geneticists to increase the throughput of phenotyping. With clear evidence of accurate estimations for different properties including leaf nutrients, chlorophyll, mass, protein, starch, sugars, water content, metabolites, and temperature (Silva-Perez *et al.*, 2018; Yendrek *et al.*, 2017; Ely *et al.*, 2019; Ge *et al.*, 2019; Cotrozzi *et al.*, 2020; Chai *et al.*, 2021), trait measurements across hundreds of plants of different species within a short period of time at a low cost is becoming more realistic. This is highly beneficial for quantitative genetics and can significantly accelerate the process of identifying genes and genetic markers controlling a specific target trait while decreasing costs. In these applications the model accuracy and robustness are immensely important. The development of robust models using hyperspectral data is often challenging, especially in situations where models have to be transferred from one dataset to another due to the lack of sufficient numbers of samples. Grzybowski *et al.* (2021) have demonstrated that model accuracy markedly diminishes when transferring models from one year to another even when using data from the same crop species, grown in the same region, and collected by the same research group. This can hinder the use of hyperspectral data for plant phenotyping. However, as shown in our study, ‘extra-weighted spiking’ can provide an effective solution by improving model transferability across multiple species or growing conditions. As only a minimal number of samples are required, the substantial improvements in model performance should frequently outweigh the modest increase in cost and time invested in phenotyping efforts.

## Conclusions

In this study, we have reported a leaf-level VIS-NIR-SWIR spectral library that can potentially be used for high-throughput phenotyping of several leaf physiological and biochemical traits. A comparison of two machine-learning modeling techniques showed the superiority of PLSR to DNN. Models calibrated on the spectral library showed poor transferability to external test datasets containing different plant species. However, extra-weighted spiking with a small number of samples (*n*=20) from external datasets markedly improved model transferability, indicating that this technique can be effectively deployed to improve the use of spectral libraries under diverse conditions and to widen the applicability of VIS-NIR-SWIR data in plant phenotyping. Overall, our study has shown that VIS-NIR-SWIR leaf spectral libraries can enable rapid and low-cost analysis of several important leaf physiological and biochemical traits. In addition, we have validated the analytical approach of extra-weighted spiking as a means to extend the applicability of the spectral library for more species and experimental conditions.

## Supplementary data

The following supplementary data are available at *JXB* online.

Table S1. Descriptive statistics of the nine leaf properties in the spectral library and the independent, external test sets.

Table S2. Prediction statistics of the different modeling schemes on different leaf properties and using two modeling techniques.

erad129_suppl_supplementary_tables_S1-S2

## Data Availability

All data including the VIS-NIR-SWIR spectral library used in this study are available from the corresponding author, Yufeng Ge, upon request.
